# Is Male Rheumatoid Arthritis an Occupational Disease? A Review

**DOI:** 10.2174/1874312901711010088

**Published:** 2017-07-27

**Authors:** Dan Murphy, David Hutchinson

**Affiliations:** 1 Rheumatology Department, Royal Cornwall Hospital, Truro, Cornwall TR1 3LH, UK; 2 University of Exeter Medical School, Cornwall Campus, Knowledge Spa, Truro, Cornwall, TR1 3HD, UK; 3 St. Austell Healthcare Group, Wheal Northey Surgery, St Austell, Cornwall, PL25 3EF, UK

**Keywords:** Silica, Dust, Occupation, Inhalation, Cadmium, Cigarette smoking, Bronchial associated lymphoid tissue, Adsorption, Rheumatoid arthritis

## Abstract

**Background:**

Rheumatoid arthritis (RA) is a systemic, inflammatory disease with an estimated global prevalence of 0.3–1.0%. An unexplained association exists between low formal education and the development of RA independent of smoking. It is established that RA is initiated in the lungs and that various occupations associated with dust, fume and metal inhalation can increase the risk of RA development.

**Objective:**

The objective of this review is to evaluate published clinical reports related to occupations associated with RA development. We highlight the concept of a “double-hit” phenomenon involving adsorption of toxic metals from cigarette smoke by dust residing in the lung as a result of various work exposures. We discuss the relevant pathophysiological consequences of these inhalational exposures in relation to RA associated autoantibody production.

**Method:**

A thorough literature search was performed using available databases including Pubmed, Embase, and Cochrane database to cover all relative reports, using combinations of keywords: rheumatoid arthritis, rheumatoid factor, anti-citrullinated peptide antibody silica, dust, fumes, metals, cadmium, cigarette smoking, asbestos, mining, bronchial associated lymphoid tissue, heat shock protein 70, and adsorption.

**Conclusion:**

We postulate that the inhalation of dust, metals and fumes is a significant trigger factor for RA development in male patients and that male RA should be considered an occupational disease. To the best of our knowledge, this is the first review of occupations as a risk factor for RA in relation to the potential underlying pathophysiology.

## 1 INTRODUCTION

### 1.1 Background

Rheumatoid arthritis (RA) is a common autoimmune inflammatory disease with an estimated global prevalence of 0.3–1.0% [1], and primarily targets the small joints of the hands [2]. The overall cost to the individual with RA as a consequence of fatigue, joint inflammation and the subsequent development of joint deformities are impaired physical functioning, reduced work productivity and activities of daily living [3]. The overall economic cost of RA to society is substantial. A study of RA patients in Norfolk UK with longstanding disease of 10-15 years reported an annual cost of approximately £3000 per patient (2013 prices) for direct health care alone [4]. Therefore studies identifying potential causes of RA that are avoidable and are of huge importance to at risk individuals and also wider society.

Literature has established the lung as a primary site for disease development [5], although the exact aetiology remains unclear. Whilst genetic susceptibility plays a role, concordance rates of 9.1% (95% CI 1.9 to 24.3) in monozygotic twins have been found [6] suggesting that environmental factors are of great importance regarding the pathogenesis of RA.

 Cigarette smoking has emerged as an important risk factor for RA, with a strong association between ever smoking and RA in monozygotic twin pairs demonstrating an odds ratio (OR) 12.0, (95% CI 1.78–513) [7]. Smoking, as the most important environmental trigger factor for RA, associates with rheumatoid factor (RF) and antibodies to citrullinated peptide antigens (ACPA) rather than RF and ACPA negative RA [8].

 Further inhaled environmental risk factors for RA have been suggested including exposure to silica [9-13], construction work [13-15], asbestos [16, 17], mineral oils [18, 19] farming and pesticide exposure [14, 16, 19, 20], electrical and electronics work [14, 15], textiles [21] and roadside dust [22, 23]. Specific occupations have been associated with ACPA positive RA [14, 15, 18, 20, 22].

 Citrullination of proteins in the lungs, as found in the longevity-associated RA risk identified in cigarette smoke inhalation [24], could equally be applied to other inhalational exposures [25]. Such exposures predominantly relate to occupation in male populations, though evidence exists linking RA risk to female occupational inhalation, such as recently seen in textile workers [21] and inhalational exposures in the wider environment [20, 22, 25, 26].

### 1.2 Key question

Why do people exposed to workplace dust or fumes in addition to cigarette smoke demonstrate a greatly elevated risk of RA?

### 1.3 Hypothesis

In short, we suggest that RA should be considered an occupational disease as a consequence of dust and fume inhalation in the workplace, as such exposures stimulate immune tolerance breakdown. Furthermore, these exposures form a lung substrate that can adsorb heavy metals from other environmental sources such as cigarette smoke.

We argue that metals have the potential to induce lymphoid tissue in the lung, generating ACPA and RF production locally. We highlight the process of adsorption as critical for RA disease development in men and discuss the pathophysiology of autoantibody generation resulting from occupational inhalation in stimulating RA disease development.

### 2 METHODS

A literature search was performed using Pubmed, Embase, and Cochrane databases and to cover all relative reports. Titles and abstracts of identified articles were screened for eligibility and relevance to the key question and a priori hypothesis generation as detailed above. Duplicates and reports generated that were not relevant to the study aims were removed. Reference lists of relevant articles were searched for further evidence by hand around mechanisms of action and further hypothesis refinement.

### 2.1 Occupational Exposure

Initial searches were conducted using the following keywords: “occupation OR mining OR silica”, in combination with disease specific term “Rheumatoid arthritis”. Subsequent searches of disease and exposure detail were made using the terms “Rheumatoid factor OR anti-citrullinated protein antibody/ies”, AND “silica OR dust OR fumes OR metals OR cadmium OR asbestos OR mining”. A separate search was made using the combination terms “rheumatoid AND smoking AND occupation”. Following hypothesis generation and in the process of refinement, further searches were made using the combination terms “Rheumatoid arthritis AND bronchial associated lymphoid tissue OR adsorption OR heat shock protein 70”.

### 2.2 Appraisal

For occupational exposures, studies were examined for information relevant to output fields of sex, country of study, follow up period and exposure type. Reported or calculable RA risk being statistically significant for increased RA risk (with lower 95% confidence interval greater than 1.0) was used for inclusion.

### 2.3 Integration

For critical review purposes, statistically significant data pertaining to increased RA risk in male occupational exposures was integrated into a single Table **1**, for descriptive comparison. Data not reaching statistical significance was not included in the table, though elements of non-statistical significance are included in the discussion. Heterogeneity in study populations, comparison groups and outcome measures precluded further pooled analysis or systematic review.

## 3 INHALATIONAL WORKPLACE EXPOSURES

### 3.1 Mining/ Quarry Workers

Mining has long been associated with RA [27]. A cohort follow up study of 1026 Finnish granite quarry and processing yard workers from 1940-1981, demonstrated RA incidence rate ratio (defined as award to disability pension for RA), over five times higher than expected, compared to general population statistics. Silica exposure was postulated as the primary mechanism of aetiology [9]. Interestingly, RA was seen in the absence of frank silicosis, suggesting silicosis per se does not account for the observed increased risk of RA and an alternate pathophysiology is likely.

Stolt *et al*. [11] found elevated ACPA+ RA risk in silica exposed Swedish quarry workers compared to unexposed controls (OR 1.67, 95% CI 1.13–2.48), but no increased risk of ACPA- RA when compared to unexposed controls. This was particularly evident amongst men exposed to stone dust and rock drilling. In terms of outcome, Swedish miners and quarry workers have also demonstrated the highest and most consistent standardised incidence ratios (SIR) across three cohorts of patients hospitalised due to RA (SIR 1.4, 95% CI 1.0-1.9 for 43 RA miners as per 1960 and 1970 census data) [14].

An association was seen in mortality odds ratio between postulated silica exposure and RA (OR 1.19, 95% CI 1.12-1.25) on analysis of death certificate data from 27 USA states from 1982-1995 [12]. Comparing 15/1237 RA silicotics with 20/6185 RA controls, this study was limited by the death certificate record of occupation rather than complete occupational history, and diseases/conditions contributing to cause of death. Categorisation of silica exposure was made based on occupation type rather than duration or intensity of exposure. However, stringent exclusion criteria would suggest bias towards the null hypothesis, suggesting that the significant risks presented may be an underestimate.

Mining has long been associated with extra-articular RA manifestations such as pulmonary nodules. Caplan’s syndrome, first described in 1953, classically occurs in coal dust exposed RA cases, characterised by development of pulmonary nodules 0.5-5cm throughout the lung field, distinct from silicosis [27]. Given that the lung is an important site of RA initiation [5], this is of contemporary interest in RA aetiology. Radiological changes of Caplan’s syndrome can precede RA development, and regional differences in the incidence of Caplan’s syndrome in coal miners have been noted [28]. Underground coal miners in Staffordshire, UK, demonstrated a significantly increased risk of RA development (OR 8.47, 95% CI 2.59 to 27.66) [29]. Workers exposed to Kaolin (China clay) dust demonstrate the highest prevalence of Caplan’s syndrome ever reported, 12 times higher than the prevalence noted in coal dust associated pneumoconiosis claimants (2/68, 3% [30] vs. 37/14000, 0.26% [27]). This raises intriguing questions as to the common components of inhaled dust responsible for RA pulmonary nodule development, and suggests that both dusts are implicated in RA development.

### 3.2 Construction Workers

Elevated RA risk has been demonstrated amongst construction workers. Li **et al**. found significantly elevated risk amongst three cohorts of general construction workers studied, with SIR of 1.4 (95% CI 1.2-1.6), for RA hospitalisations 1964-2004 comparing to census data for occupations over census periods covering 1960-1970 [14]. No elevated SIR was seen amongst woodworkers or bricklayers. A recent abstract on the Epidemiological Investigation of Rheumatoid Arthritis (EIRA) study of 3295 new RA cases in defined Swedish geographical areas compared to 4912 controls, suggested an OR of 2.6 (95% CI 1.3-4.9) for bricklayers and concrete workers [15]. However, this abstract does not detail actual numbers in the trades listed, and whilst the authors report that results are adjusted for smoking, no methodology is given. Furthermore, the authors included patients aged 18-70, and only report “last occupation” listed. Given the longevity of risk from the known inhalational insult of smoking [24], some acknowledgement of risk longevity conferred by other inhalational exposures would be expected. With the notable exception of mineral oil exposure, polarisation of RA risk estimates occurs when a latency period is used in occupational exposures [18], indicating a long induction period between exposure and RA development. Ergo, an upper age cut off of 70 years may miss those occupationally exposed cases over the age of 50, who may not present until well into their 70s and crucially will not account for multiple occupational co-exposures.

Asbestos exposure, commonly found in historical building trade populations, was found to be an RA risk factor amongst 12/74 incident cases compared to 19/382 referents in multivariate analysis of lifelong occupational exposures (OR 2.5, 95% CI 1.0-6.8) [16]. Further evidence is found in a case-control study of community residents and workers exposed to asbestos contaminated vermiculite in Montana, USA, who demonstrated a strikingly elevated RA risk in those aged over 65 (OR 3.23 (95% CI 1.31-7.96) [17]. Interestingly, no significant increased risk was seen in those aged under 65, further highlighting the need to acknowledge a latency period between exposure and disease development.

### 3.3 Electrical Workers and Workers Exposed to Metal Fumes

Electrical workers demonstrated a non-significant, though consistent elevated RA hospitalisation risk (OR 1.1-1.3, (95% CI 1.0-1.3) [14]. Ilar *et al*. suggest an OR of 1.8 (95%CI 1.0-3.1) for ACPA+ RA, and OR 2.1 (95%CI 1.1-4.0) for ACPA- RA, amongst electrical and electronics workers [15]. In the UK, electricians represent a subset of construction industry workers with multiple co-exposures. Inhalation of silica, non-silica inorganic, and organic dusts from physical interactions with building industry substrates. Activities such as drilling and chiselling out concrete, wood and gypsum plasterboard are likely to be relevant. Further specific aspects to electrical work involve handling and assembly of componentry that exposes workers to metal fumes. Toxic elements (such as cadmium) have been sequentially reduced and eliminated from solder, plastics and brazing fillers in modern European applications [31]. However, a longitudinal study of ten workers exposed to silver solder in the UK demonstrated the longevity of historical heavy metal fume exposure [32].

Other direct metal fume exposed trades demonstrate increased RA risk. An increased RA risk was reported in 29/74 Swedish mechanics, sheet metal workers and welders (OR 1.8, 95% CI 1.0-3.4) [16]. A single cohort of Li *et al*’s study [14] showed a significantly elevated SIR of 1.2 (95%CI 1.1-1.4) amongst foundry workers, with two large cohorts of mechanics and metal workers demonstrating slight, though insignificant, SIR of 1.1 (95%CI 1.0-1.1) and 1.0 (95% CI 1.0-1.1) respectively. Ilar *et al*. [15] suggest an OR of 2.6 (OR 1.0-7.4) for ACPA+ RA in smelters and metal foundry workers. A retrospective cohort study of workers recycling scrap metals at an electrical arc furnace in Italy noted 3/331 incident cases of RA over a 20 year follow up period, a dramatically increased RR of 6.18 (95%CI 2.00-19.02), when compared to 420/20332 incident RA cases in the same town over the same period [33]. Despite the relatively small sample size and lack of directly comparable working group, this cohort demonstrated an appreciably elevated RA risk, thought due to foundry dust exposure containing a range of potential toxins including heavy metals [34].

### 3.4 Mechanics and Workers Exposed to Mineral Oils

In an analysis of 281 cases to 507 referents aged 25-75 years presenting to a single secondary care hospital in southeast Sweden, an increased though non-significant RA risk was seen amongst machine and engine repairers (OR 2.1, 95% CI 0.8-5.6), with specific exposure to hydraulic oils demonstrating an OR of 1.8, (95% CI 0.7-4.3) [35]. Li *et al.* found that engine and motor operators demonstrated consistent though insignificant SIR elevation across three cohorts (SIR 1.1-1.2, 95% CI 1.0-1.5 combined) [14].

A report on the EIRA study compared 135 RA males exposed to different types of mineral oil to 132 controls, comparing seropositive and seronegative RA [18]. Exposure to any mineral oil demonstrated an increased risk (OR 1.3, 95%CI 1.0-1.3), with motor oil exposure (OR 1.2, 95%CI 0.9-1.8) carrying higher risk than others. When subdivided by autoantibody positivity, significantly increased risk to any mineral oil exposure was only seen in seropositive disease (ACPA+ OR 1.6, 95%CI 1.1-2.2). No significant increased risk for asphalt exposure was seen, unlike findings elsewhere (OR 14.0, 95%CI 1.2-99.9), albeit in limited numbers (4 cases: 1 referent) [16]. Limited correction for smoking was made via “ever” and “never” smoker categories. The authors noted a possible interaction between smoking and mineral oil exposure, but report an inability to reach a firm conclusion due to small numbers (ACPA+ RA attributable portion due to interaction= 0.5, 95%CI -0.2-1.2). No attributable portion was reported for RF+ RA.

### 3.5 Farm Workers

Farming has long been associated with increased RA risk. A small but persistent SIR was noted in 821 RA farmers in Li *et al's* combined cohort as per 1960-1970 data (SIR 1.1, 95% CI 1.1-1.2) [14]. A further study of incident cases in Sweden found an OR 2.4 (95% CI 1.1-5.2) amongst 20 farm worker RA cases to 41 referents [16]. Interestingly, this study examined potential exposures which farm workers may encounter. On multivariate analysis with adjustment for age and smoking, no statistically significant increased risk was found for exposure to pesticides, farm animals, mineral oils or organic dusts. However, subsequent pooled analysis of specific occupational exposures with at least 50 exposed subjects amongst 176 cases and 630 referents, with adjustment for age and smoking, showed statistically significant increased risk amongst those exposed to crops and forage (OR 3.2, 95% CI 1.6-6.7 for >20 years exposure), and for fertilisers (OR 3.0, 95% CI 1.3-3.8). No dose-duration relationship was observed.

A longitudinal American study of 23570 spouses of pesticide license applicants from 1993-1997 found no association of RA with growing up on a farm or years living on a farm, on structured follow up interview in 2010 [19]. Detailed exposure analysis of 271 RA cases revealed increased risk from lifetime pesticide use (OR 1.4, 95% CI 1.0-1.6), and application of chemical fertilizers (OR 1.7, 95% CI 1.1-2.7). Amongst specific pesticides examined, an elevated but non-significant risk was seen for DDT (OR 1.9, 95% CI 0.97-3.6). Significant risks were seen for maneb/ manecozeb fungicides (OR 3.3, 95% CI 1.5-7.1), and chemical fertilizers (OR 1.7, 95% CI 1.1-2.7).

## 4 DISCUSSION

**Fig. (2):**
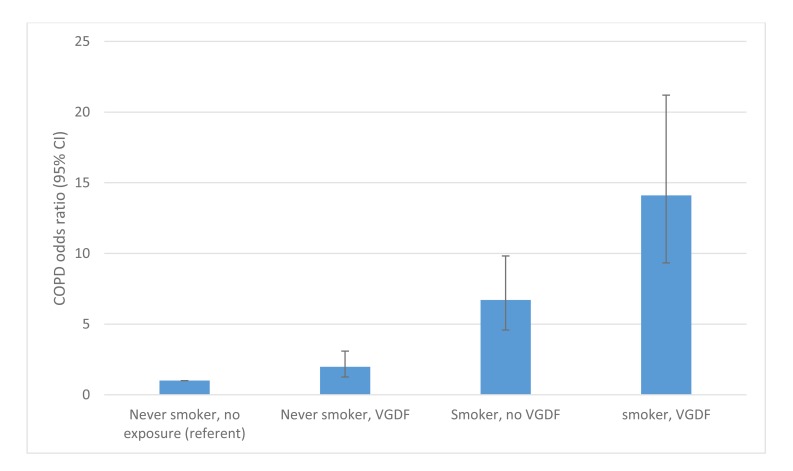
COPD risk: interaction between smoking and vapour/gas/dust/fume (VGDF) exposure. Adapted from Blanc *et al* [40].

Low formal education has consistently been found to be associated with RA [36-38]. All the occupations listed in Table **1** require no formal academic qualifications and it is noteworthy that no study as yet has considered an individual’s occupation as a confounding factor when considering low formal education levels as a risk factor for RA development. Likewise the stark contrast in mortality reported by Pincus *et al*. [39] in RA patients over a nine year period with a low formal education (45% mortality) compared to those with the highest formal education (5% mortality) has not been explained. However, occupations undertaken by working class individuals that expose them to vapours, gas, dust or fumes greatly increases the risk of chronic obstructive pulmonary disease (COPD) particularly in smokers, with an OR 14.1 (9.33-21.2) compared to never smokers with no such work exposures [40]. The “double-hit” of occupational exposure and smoking combined risk was far higher than the OR of 6.71 (4.58-9.82) in those smokers without such work exposures (Fig. **2**).

Whist the contribution of occupational exposures to chronic obstructive airways disease has been acknowledged [41], these occupations demonstrate only modest odds ratio rises in non-smokers. This is exemplified by Blanc’s study where significant but only modest differences were seen in never smokers when combined into broad categories of vapours, gas, dust and fume exposure in the longest held occupation (OR 1.98 (1.26-3.09) [40]. The impact of these exposures becomes particularly apparent when occurring in combination with smoking Fig. (**2**). This phenomenon is highly relevant to RA as men who work in dusty trades are highly likely to have smoked as the vast majority of male RA patients have been smokers (77%) [8].

Occupational risk alongside smoking delivers a substantial “double-hit” for disease development. For example, silica dust exposure and smoking combined confer an increased risk of ACPA positive RA [11, 15, 18]. Interestingly, a pronounced risk interaction of silica dust and cigarette smoking for ACPA+ RA development was seen amongst co-exposed workers: silica only, OR 1.15 (95% CI 0.42-3.15); current smoking, OR 2.78 (95% CI 1.77-4.38); silica + current smoking OR 7.36 (95% CI 3.31-16.38), rising to OR 14.9, (95% CI 5.32-37.84) for >20 pack years smoked Fig. (**3**). This risk exceeded the expected separate effects of silica exposure and current smoking, indicating an interaction between these exposures (attributable proportion due to interaction = 0.60 95% CI 0.26-0.95). No explanation for this important interaction has been proposed in the literature.

Recent analysis of 240,983 construction industry workers in Sweden suggested that “other inorganic” dusts are independent RA risk factors, in addition to silica [13]. Among ever smokers, both silica and other inorganic dust exposure were associated with increased RA risk: RR 1.36 (95% CI 1.11-1.68) and 1.42 (95% CI 1.17-1.73) respectively. However, no increased risk was seen amongst dust-exposed never smokers Fig. (**4**). This poses interesting questions, both as to the risk posed by inorganic dusts other than silica, and to the pathophysiology of the risk interaction of dust and cigarette smoke co-exposure.

Here we suggest that silica and non-silica inorganic dusts act as an adsorber of heavy metals entering the lung *via* cigarette smoke. Cigarette smoke contains the metals aluminium, cadmium, chromium, copper, lead, manganese, mercury, nickel, selenium, vanadium and zinc. Cadmium has emerged as the most important of these metals in terms of increased levels relative to non-smokers and the associated increased risk of cardiovascular disease and COPD [42].

It has been hypothesised that inhalation of the metal cadmium links the established risk of smoking for RA and many of the occupations demonstrated to have an elevated RA risk [43]. Recent South Korean literature reports a significant RA OR rise of 1.62 per 1µg/L increase in serum cadmium, and a smaller significant OR rise in serum lead [44]. Importantly cadmium has recently been demonstrated to citrullinate intracellular cytokeratins [45], which may potentially lead to immune tolerance breakdown and ACPA generation. Furthermore in micromolar concentrations (1–10 μM range), cadmium is associated with a pro-inflammatory state [46], and high dose administration to Wistar rats has been shown to exacerbate collagen induced arthritis disease development, demonstrating pro-inflammatory cytokine expression [47].

## 5 LIMITATIONS OF EVIDENCE

Over the course of an occupational lifetime, many workers will be exposed to multiple agents. For example, in less skilled labouring construction workers, co-exposure to silica dust, inorganic non-silica dust and wood dust is common. This is particularly true in poorer populations, where trade sub-specialisation and adherence to health and safety legislation will be less evident. As previously described, lower socio-economic status is linked with RA [36-38]. Given the longevity of increased risk for RA found in smoking [24], occupational exposure to dust or fumes may not present until long after exposure cessation.

Therefore, studies may be confounded by multiple inhalational insults, even when correcting for smoking. Blanc *et al*. [13] excluded 40645 workers who were exposed to wood dust, gas or fumes to reduce confounding in their analysis of silica *vs*. non-silica dust exposed construction workers. In the above study, capturing data from occupational health service records for unionised construction workers existing from 1968-1993, culminated in enrolment of approximately 80% of the eligible workers. Analysis of the “missing” 20% and the excluded, co-exposed workers would be interesting; one would suggest predominance towards lower socio-economic status groups. Such workers are more likely to reside in unskilled “itinerant” trades, and thus become excluded from analysis. Non-unionised workers are not mentioned, which may have limited relevance in Swedish construction but is highly relevant when generalising findings elsewhere. We suggest many co-exposed cases of interest may have been excluded, underestimating risk.

As with construction workers, farmers perform a heterogenous mix of tasks in their work which can result in inhalation exposures. Soil type, rainfall and wind patterns affect aerosolisation of particles. Oil exposures from machinery vary, along with solvent, concrete, cement and general building dust exposure from the variety of tasks that some farmers perform. Metal fumes from welding have been associated with increased RA risk amongst spouses of farmers [20]. We have previously suggested that heavy metal-laden particle inhalation may be an RA risk factor for farmers using pesticides and fertilisers [26]. Maneb and mancozeb fungicides are approximately 21% manganese by weight. Their use associates with increased levels in residential house dust, with concentrations declining with distance from use. Long-term Mancozeb use has shown evidence of wider environmental contamination [48]. Additionally, cadmium exposure from phosphate fertiliser may account for the RA risk seen in farmers. Rates of cadmium contamination in soil can range from 0.3 to 1.2 g/ha from contaminated fertiliser application [49], though this specific risk is largely of historical significance due to controls on the use of such fertilisers. Detailed information of exposure type and duration and intensity is needed to refine the potential risks that farmers face.

Swedish case referent studies utilising census defined occupations and hospitalisation records dominate the literature for occupational risk in RA. Whilst illuminating, such studies do have limitations. Patients in manual jobs may present to hospital more readily as they may be unable to complete tasks that would not present a problem in sedentary occupations, leading to possible risk overestimation in hospitalisation data. Symptomatic RA patients pre-census may have already switched from “heavier” manual occupations to lighter trades, whilst similarly symptomatic patients in those lighter trades may have been able to continue in the same occupation. As such, single cohort registry data may underestimate risk in heavier, manual trades, and overestimate risk in lighter occupational categories in which working conditions can be adapted to meet the needs of RA patients. Comparing occupation and socioeconomic status based on historical census occupation data, Li *et al*'s [14] study was limited to those requiring hospital treatment, therefore misses non-hospitalised disease occurrence. Linking RA hospitalisation to preceding census occupation may reflect selection bias towards ill health, and is a criticism that can be levelled at all large cohort surveys measuring disease incidence via hospitalisation records. In having a cohort of patients who held the same job in 1960 and 1970, this study includes patients who held the same occupation over a ten-year period, and may mitigate against occupational selection bias. Given these caveats, one may expect sedentary occupations to predominate, and the mix of active technical and mechanical occupations seen here and elsewhere is therefore likely to be an underestimate. Demonstrating elevated RA risk over different census reference periods, a strong argument can be made for miners, engine and motor operators, construction workers, electrical workers and farmers having an increased risk of RA.

Extrapolating Swedish data to represent populations around the world has limitations. Cigarette smoking in Sweden has a fascinating reverse social class gradient on analysis of 55,000 participants in the 1960 census, with both long term and heavy smoking being twice as common amongst non-manual workers as manual workers, and ten times more common than in those working in farming and agriculture [50]. This trend is opposite to the UK, where smoking has consistently shown a lower socio-economic class predominance; in 1982, 49% unskilled working men smoked, compared to 20% non-manual professionals [51]. Inhalational co-exposure to dust and cigarette smoke is therefore less likely in the Swedish populations reported than in UK equivalents.

## 6 PATHOPHYSIOLOGIC CONSIDERATIONS OF VAPOUR, GAS DUST AND FUME INHALATION IN RELATION TO RA DEVELOPMENT

### 6.1 Inducible Bronchial Associated Lymphoid Tissue (iBALT)

The lung is now considered a principal site for the development of ACPA and RF positive RA [5]. A study of established RA demonstrated evidence of iBALT more commonly than in inflammatory lung disease without RA [52]. iBALT has the appearance of ectopic lymphoid follicles similar to those observed in rheumatoid joints [53]. iBALT contains numerous B cell follicles containing germinal centres and follicular dendritic cells. Lymphocyte lung infiltration was more frequently found in ACPA-positive RA patients (50%) as compared with ACPA-negative RA patients (17%) and controls (13%). Critically germinal centres, B cells and plasma cells were only found in the lungs of ACPA-positive RA patients [54].

Cigarette smoking is strongly associated with the development of iBALT as the expression of iBALT was significantly more common in smokers than non-smokers (82% (14/17) v 14% (2/14) respectively) [55]. Furthermore silica has also been observed to generate iBALT in a murine model [56]. Rheumatoid pulmonary nodules have also been observed to contain lymphoid aggregates containing B lymphocytes and, in some cases, demonstrate characteristic features of lymphoid follicles [57]. The initial histological examination of the rheumatoid pulmonary nodules in the lungs of Welsh miners with Caplan’s syndrome eluded to the presence of germinal centres with lymphoid collections in the outer collagen layer [58], which have never been described in peripheral nodules [59]. Pulmonary rheumatoid nodules, therefore, have the potential to generate rheumatoid associated autoantibodies and it is unsurprising that pulmonary nodules have been noted to precede the development of RA, invariably being associated with seropositive rather than seronegative disease [60]. For example, the presence of pulmonary rheumatoid nodules in Welsh coal miners who had no history, signs, or symptoms of RA was associated with a high prevalence of positive rheumatoid factor tests (60%). The potential importance of pulmonary nodules may have been over looked as on plain chest x-rays lung nodules were present in only 0.3% of RA patients [61]. However a study utilising CT imaging of the lung demonstrated pulmonary nodules in 22% of RA patients and subpleural micronodules and/or pseudoplaques in a further 17% of patients [62]. We suggest that accumulation of metals in the lung may predispose individuals to rheumatoid pulmonary nodules. The histology of a rheumatoid nodule is that of a granuloma with a palisade of cells proven to be a dual population of macrophages and fibroblastic cells clustered around a central necrotic core of fibrin [63]. The macrophages are activated and have the appearance of epithelial cells and are termed epithelioid histiocytes [64]. Occasionally these histiocytes coalesce to form multinucleated giant cells in the rheumatoid nodule. Metal inhalation has been reported to be associated with the development of pulmonary macrophage derived multinucleated giant cells [65]. Metals such as beryllium are also linked to the formation of pulmonary nodules with histological features similar to rheumatoid nodules and can cause a chronic pulmonary granulomatous disease termed berylliosis which may arise long after exposure has ceased with a reported latency period of up to 40 years [66]. Raised levels of the heavy metals cobalt and chromium are associated with complications associated with metal on metal hip replacements. One of these complications includes the formation of tertiary lymphoid tissue around the implant and this tissue is identical to that observed in joints of RA patients [67]. There appears to be an association between significant bodily levels of metals such as copper and cadmium in RA independent of smoking [68, 69]. These data suggest that RA patients are exposed to metals and that heavy metals can induce the development of ectopic lymphoid tissue.

Further animal model evidence strengthens the “double-hit” hypothesis in terms of granuloma formation. Rat lung instilled with cadmium-containing silica nanopaticles demonstrated greater expression of pro-inflammatory cytokines and granuloma formation than lung exposed to cadmium alone or silica nanoparticles alone [70]. Nanoparticle use per se may be of interest as an evolving occupational risk, as inhaled silica and carbon nanoparticles, aerosolised in electronics componentry and production of lightweight materials, can induce lung citrullination and activate peptidyl arginine deaminase [71]. Given that numerous metals are found in cigarette smoke [42], one possible explanation for the association between cigarette smoking and iBALT development is the accumulation of metals in the lungs of smokers; a process likely to be accentuated by the presence of various occupational dusts.

### 6.2 Enhanced ACPA Generation

It is well documented that the generation of ACPAs occur prior to the development of RA and greatly increase the risk of disease development [72]. Examination of bronchoalveolar lavage fluid from RA detected ACPAs and the levels of these antibodies were increased in patients that had more well-developed iBALT [52]. These data suggest that ACPA antibodies are produced locally in the lung by plasma cells contained within iBALT. In addition to silica and cadmium inducing iBALT, both nanoparticles have been observed to citrullinate intracellular proteins such as cytokeratins [45, 71]. In the case of silica, citrullination occurred via the peptidylargininedeiminase (PAD)-dependent mechanism [71].

### 6.3 Rheumatoid Factor Production

Rheumatoid factor is an autoantibody directed against the rheumatoid binding site of the Fc of immunoglobulin G (IgG) [73]. Rheumatoid factor positivity is associated an enhanced risk of RA development [74]. Rheumatoid factor is more frequently observed in non-RA smokers [75], and individuals with lung disease associated with exposure to coal dust [60], asbestos [76] and silica [77]. Revealing the processes that result in RF generation may prove insightful for understanding how cigarette smoke and industrial inhalational exposures increase the risk of RA development.

Newkirk *et al*. [78] described a mechanism by which cigarette smoke induces either IgM RF or IgA RF to be generated by B cells. This mechanism is dependent on the generation of an IgG immune response to heat shock protein 70 (HSP 70). Complexes of IgG-HSP70 double bind to RF expressing B cells via an interaction with the B cell receptor and CD91 with the subsequent production of IgM RF and IgA RF [78]. Therefore, environmental insults that associate with the induction of HSP 70 with a secondary IgG response are likely to be important with regards to the generation of IgM RF and IgA RF. HSP 70 expression is enhanced in the rheumatoid joints and an IgG autoantibody response to HSP 70 is significantly raised in recent onset RA (53.0%) compared to normal controls (4%) [79]. Interestingly exposure to dusts alongside heat and noise in the work place can generate a significantly enhanced immune response to HSP 70 (40%) compared to office workers (19%) [80]. Likewise in smokers of North American Indian origin (an ethnic group with a very high risk for RA) there is a reported significantly increased prevalence of IgG anti-HSP70 positive individuals (40%) as opposed to non-smokers (5%) [78]. Cadmium and other metals (copper and mercury) are strongly linked to the upregulation of HSP 70 [81], as is silica [82]. No studies to date have correlated RF levels in RA with dual exposures to various dusts and cigarette smoke and the relationship between RF and IgG HSP 70 autoantibodies.

### 6.4 Adsorption in Dust Exposure

All the occupations discussed involve inhalation of dust, fumes or particles, with the potential for adsorption of toxic elements such as heavy metals, from concurrent environmental co-exposure (most commonly through cigarette smoke). This process is seen in, but not exclusive to, silica dusts, with adsorption of toxic heavy metals such as cadmium directly onto the intra-pulmonary substrate inhaled previously, dramatically increasing total body levels. We suggest a hypothesis of adsorption of trace elements in vitro onto previously inhaled substrates as a cause of increased, interactive RA risk seen in sequential inhalational exposures. Cadmium has been described as the most important toxin in inhaled cigarette smoke [42]. Adsorption explains the pronounced interaction of silica dust and current smoking >20 pack years co-exposure amongst exposed workers (OR 14.9, 95%CI 5.32-37.84, Fig. (**3**)), described by Stolt [11], and the attenuation of risk seen by Blanc [13], in silica and non-silica dust exposed never smokers.

Quantification of the exact adsorption capacity of dust varies. In laboratory analysis of a single known adsorption substrate, sorption depends heavily on experimental conditions such as pH, metal concentration, ligand concentration competing ions, and particle size. This is before consideration of the differing adsorption capacities of multiple dusts that workers may be exposed to. For example, a general builder may inhale fine sand, gypsum, cement, and sanded wood. Occupational exposure matrices have a limited ability to capture individual variability within this due to heterogeneity of tasks and substrates, with consequent potential for misclassification bias [83].

Adsorption capability generally results from a net negative charge on structural particles of substrates, attracting and binding positively charged heavy metal particles. Binding potential is enhanced by increasing surface area, seen in finer dust particles [84]. Within mineral dusts, occurrence of trace elements depends on principal mineral species of the substrate and individual characteristics of the sorption particle. For example, bituminous coals may display a high affinity for formation of organo-metal complexes and organic acid salts, thereby potentially containing higher trace element levels in situ. Conversely, the aluminiosilicate mineral content of fine dust from anthracite coals has greater potential for further adsorption of heavy metals due to surface charges [85]. Various organic dust particles have quantifiably demonstrated adsorption of heavy metals, such as cotton, wood, wool, moss, and waste from paper and seafood industries [84]. Of non-silica inorganic dusts, commercial gypsum and industrial gypsum by-product, both widely used in the construction industry for plasters and plasterboards, have demonstrated adsorption of lead and cadmium in solution *via* sulphate binding [86]. An interesting analysis on cadmium binding via adsorption demonstrated interaction between sand, cement and clay to which construction workers would be exposed. Clay addition to sand-cement mix (a common process for enhancing mortar plasticity), exposed more ion exchange sites and altered pH, enhancing uptake capacity of cadmium via ion exchange of iron, magnesium and aluminium oxides bound in cement [87].

**Fig. (5):**
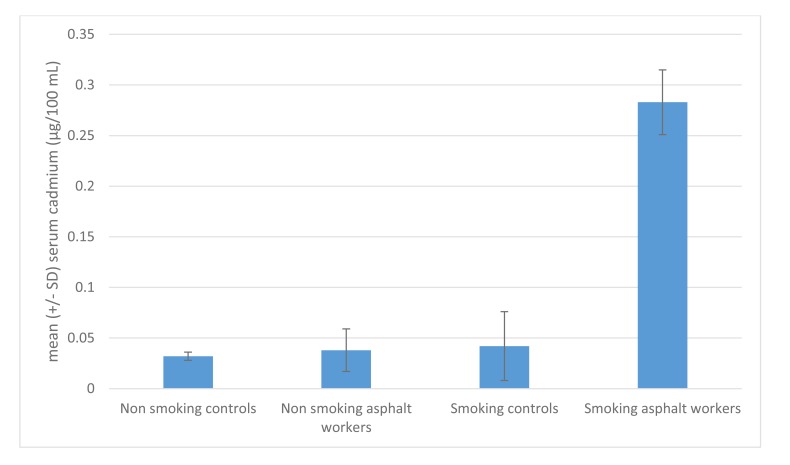
Interaction of asphalt exposure and cigarette smoking on serum cadmium levels among Turkish asphalt workers, adapted from Atasoy *et al*. [88].

We believe it is the “double hit” of inhaled particles with capacity for further adsorption from subsequent inhalation of trace elements that imparts excess risk. Most commonly, this would take the form of inhaled occupational dusts with the ability to adsorb trace elements from cigarette smoke. Further evidence for this compound exposure is demonstrated in Turkish bitumen ashphalters, who demonstrated a six-fold increase in serum cadmium compared to either non-smoking colleagues or control smokers [88] (Fig. **5**).

## 7 IMPLICATIONS FOR TREATMENT

It is well established that cigarette smoking reduces the clinical response to both methotrexate and tumour necrosis factor (TNF) inhibitors [89, 90]. Cigarette smoking is strongly associated with the titres of RF [91], and it is noteworthy that a RF titre < 20 IU/ml was highly predictive for remission or low disease activity in RA patients with established disease receiving TNF inhibitors [92]. In this study RA patients with a RF titre < 20 IU/ml were significantly more likely (OR 18.9, 95% CI 10.79-38.36) to be in remission at 12 months than RA patients with a RF titre >20 IU/ml. This is important as our own observations have demonstrated that wood dust exposed carpenters have significantly higher RF titres than non-dust exposed RA cases. Control never smokers (n=40) were noted to have RF titre of 16 IU/ml (IQR 6.7–47.2) compared to carpenter never smokers (n=8) RF titre of 86.4 (IQR 19.5–230.3), p=0.04 [93]. It is therefore conceivable that individuals who have been exposed to dust in the work place have a blunted response to methotrexate and TNF inhibitors by virtue of high RF titres, generated by inhalational insults as discussed above.

## CONCLUSION

A variety of occupations have demonstrated increased risk for RA, primarily identified through Swedish case referent and linkage studies, and involve exposure to inhalational particles, in the form of dust, fumes or both. Global health and safety legislation, working patterns and smoking habits vary considerably. Studying populations where such co-exposures are common may demonstrate further areas of enhanced RA risk.

Interaction is seen between occupational RA risk and cigarette smoking, and we present information to support a hypothesis of heavy metal adsorption onto inhaled dust particles to explain this. Further work is needed to analyse the ability of inhaled dusts to stimulate immune tolerance breakdown in the causation of RA.

## Figures and Tables

**Fig. (1):**
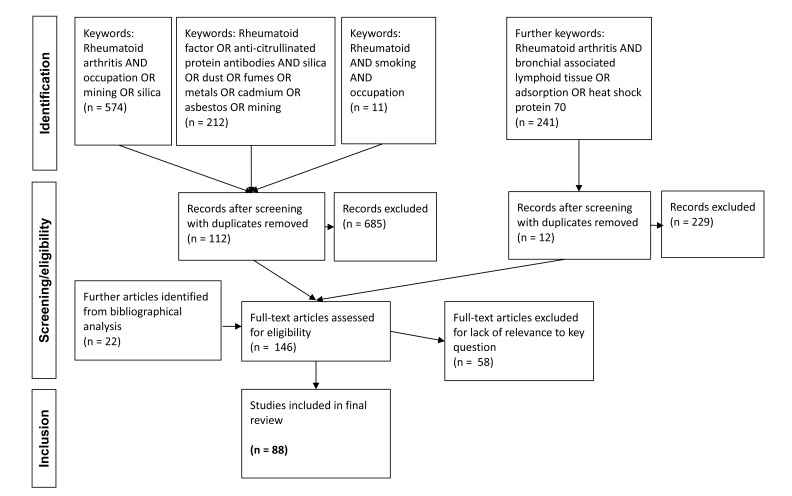
Literature retrieval flowchart.

**Fig. (3):**
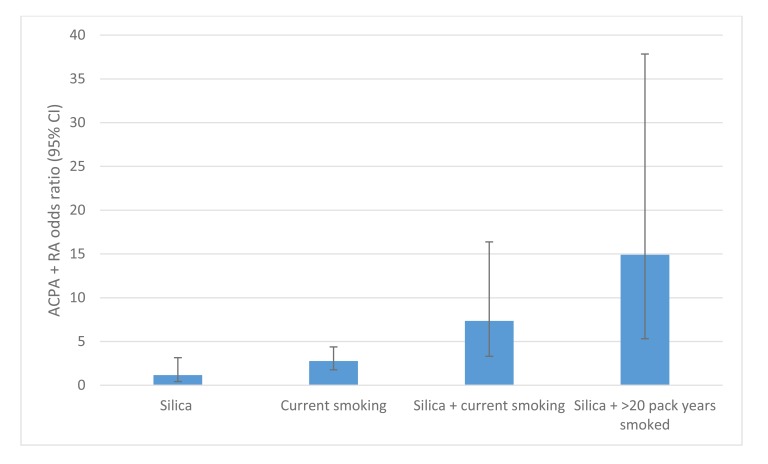
Risk interaction of ACPA+ RA in Swedish quarry workers with smoking and silica dust exposure, compared to unexposed never smoking controls, adapted from Stolt *et al* [11].

**Fig. (4):**
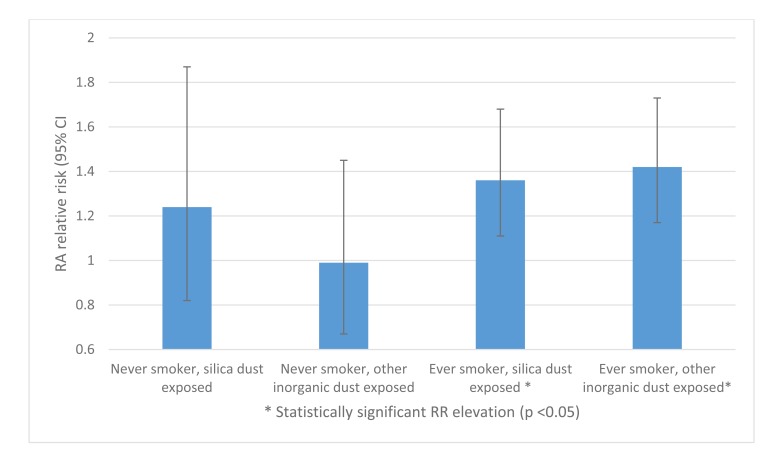
Relative RA risk: interaction between dust exposure and smoking amongst Swedish construction workers, adapted from Blanc *et al* [13].

**Table 1:** Occupational exposures previously found to demonstrate significantly increased RA risk in males.

Author(s), Publication Year and Reference No.	Country, Period of Employment and Follow–up	Study Population	Exposure Type	Cases	Controls	Reported RA risk (95% CI)	Comments
Klokars *et al* 1987 [9]	Finland 1940–1981	1026, Cohort	Granite quarry and processing yard workers	35	7.5		Comparison with age matched Finnish population statistics for all workers
Stolt *et al* 2010 [11]	Sweden 1996–2006	577 RA cases, 659 age, gender, location matched controls	Silica: stone dust, rock drilling, stone crushing	54	69	1.39 (1.13–2.48)	ACPA+ RA. ACPA– and combined RA failed to reach significance
Stolt *et al* 2010 [11]	Sweden 1996–2006	577 RA cases, 659 age, gender, location matched controls	Silica: stone dust, rock drilling, stone crushing, current smokers	21	13	7.36 (3.31–16.38)	ACPA+ RA, silica and smoking interaction
Calvert *et al* 2003 [12]	USA 1982-1995	4839231 death certificates from 27 states, silica exposed cases matched to non-silica	Silica	15/1237	20/6185	3.75 (19.2-7.32	Mortality odds ratio. OR 1.19 (1.12-1.25) following conditional logistic regression
Li *et al* 2008 [14]	Sweden 1964–2004	13820 hospitalisations for RA, cohort	Miners and quarry workers	175	-	1.7 (1.5–2.0)	Comparison with standardised incidence ratio (SIR) by occupation category in 1960 census
Li *et al* 2008 [14]	Sweden 1964–2004	13820 hospitalisations for RA, cohort	Miners and quarry workers	145	-	1.8 (1.6–2.2)	Comparison with SIR by occupation category in 1970 census
Turner and Cherry 2000 [41]	North Staffordshire, UK	6353 male pottery workers, case–referent	Underground mining	9/43	5/172	8.47 (2.59–27.66)	Referents matched on age, sex and pottery exposure
Li *et al* 2008 [14]	Sweden 1964–2004	13820 hospitalisations for RA, cohort	Construction workers	498	-	1.2 (1.1–1.4)	Comparison with standardised incidence ratio (SIR) by occupation category in 1960 census
Li *et al* 2008 [14]	Sweden 1964–2004	13820 hospitalisations for RA, cohort	Construction workers	561	-	1.3 (1.2–1.5)	Comparison with SIR by occupation category in 1970 census
Li *et al* 2008 [14]	Sweden 1964–2004	13820 hospitalisations for RA, cohort	Construction workers	210	-	1.4 (1.2–1.6)	Comparison with SIR by occupation category in 1960 and 1970 census
Ilar *et al* 2016 [15]	Sweden, unreported	3295 incident RA cases, 4912 controls	Bricklayers and concrete workers	Unreported	-	2.6 (1.3–4.9)	Abstract only publication
Blanc *et al* 2015 [13]	Sweden, 1968–1993	240983 unionised construction workers enrolled in occupational health service 1968–1993	Silica dust, ever smokers	160/52419	273/108400	1.36 (1.11–1.68)	
Blanc *et al* 2015 [13]	Sweden, 1968–1993	240983 unionised construction workers enrolled in occupational health service 1968–1993	Non–silica inorganic dust, ever smokers	202/132583	273/108400	1.42 (1.17–1.73)	
Li *et al* 2008 [14]	Sweden 1964–2004	13820 hospitalisations for RA, cohort	Electrical workers	282	-	1.2 (1.1–1.3)	Comparison with SIR by occupation category in 1960 and 1970 census
Ilar *et al* 2016 [15]	Sweden, unreported	3295 incident RA cases, 4912 controls	Electrical workers	unreported	-	2.1 (1.1–4.0)	ACPA– RA Abstract only publication
Olsson *et al* 2004 [16]	Sweden 1996-1998	74 incident male RA cases, 382 referents	Electricians, electromechanical workers, service personnel	9/74	18/382	3.4 (1.2-9.4)	
Li *et al* 2008 [14]	Sweden 1964–2004	13820 hospitalisations for RA, cohort	Smelters and metal foundry workers	271	-	1.2 (1.1–1.4)	Comparison with SIR by occupation category in 1970 census
Cappalletti *et al* 2016 [45]	Trentino, Italy 1979-2009	331 exposed workers, cohort	Electric arc furnace workers	3/331	420/20332	6.18 (2.00-19.02)	Compared to incident cases in same town over same period
Olsson *et al* 2004 [16]	Sweden 1996-1998	74 incident male RA cases, 382 referents	conductors, freight, transport workers	3/74	2/382	17.8 (1.5-207.8)	
Li *et al* 2008 [14]	Sweden 1964–2004	13820 hospitalisations for RA, cohort	Engine and Motor operators	383	-	1.2 (1.1-1.3)	Comparison with standardised incidence ratio (SIR) by occupation category in 1960 census
Svedrup *et al* 2005 [18]	Sweden 1996-2003	407 incident male RA cases, 486 controls	Mineral oil exposure	93	132/486	1.6 (1.1-2.2)	ACPA + RA, any mineral oil exposure. Specific cohort size not given for ACPA+RA
Olsson *et al* 2000 [47]	Sweden 1980-1995	102 RA cases, 248 referents	Asphalters	3/102	1/248	14.0 (1.2-799.0)	
Li *et al* 2008 [14]	Sweden 1964–2004	13820 hospitalisations for RA, cohort	Farmers	821	-	1.2 (1.1-1.2)	Comparison with SIR by occupation category in 1960 and 1970 census
Olsson *et al* 2004 [16]	Sweden 1996-1998	74 incident male RA cases, 382 referents	Farmers	20/74	41/382	2.4 (1.1-5.2)	
Parks *et al* 2016 [20]	USA 1993-2010	23570 spouses of pesticide applicants, cohort	Farming: maneb/mancozeb perticides	10/271	351/23570	3.3 (1.5-7.1)	
Parks *et al* 2016 [20]	USA 1993-2010	23570 spouses of pesticide applicants, cohort	Farming: chemical fertilisers	23/132	2540/24018	1.7 (1.1-2.7)	
